# Lipid Production of *Schizochytrium* sp. HBW10 Isolated from Coastal Waters of Northern China Cultivated in Food Waste Hydrolysate

**DOI:** 10.3390/microorganisms11112714

**Published:** 2023-11-06

**Authors:** Xiaofang Li, Xinping Yu, Qian Liu, Yong Zhang, Qiuzhen Wang

**Affiliations:** 1 Ocean College, Hebei Agricultural University, Qinhuangdao 066000, China; xiaofangli97112023@163.com (X.L.);; 2 Marine Environment Monitoring Central Station of Qinhuangdao, SOA, Qinhuangdao 066002, China

**Keywords:** thraustochytrid, *Schizochytrium*, food waste hydrolysate, initial pH, lipid

## Abstract

Marine oleaginous thraustochytrids have attracted increasing attention for their great potential in producing high-value active metabolites using various industrial and agricultural waste. Food waste containing abundant nutrients is considered as an excellent feedstock for microbial fermentation. In this study, a thraustochytrid strain *Schizochytrium* sp. HBW10 was isolated from a water column in Bohai Bay in Northern China for the first time. Further lipid production characteristics of *S.* sp. HBW10 were investigated utilizing sulfuric acid hydrolysate of food waste (FWH) from two different restaurants (FWH1 and FWH2) with the initial pH value adjusted by NaOH or NaHCO_3_. Results showed that the highest concentration of total fatty acids (TFAs) was observed in FWH2 medium with the 50% content level on the fifth day, reaching up to 0.34 g/L. A higher initial pH promoted the growth and saturated fatty acid (SFA) accumulation of *S.* sp. HBW10, achieving nearly 100% of the sum of saturated and monounsaturated fatty acids (SMUFAs) in TFAs with initial pH7 and pH8 in FWH1 medium. This work demonstrates a possible way for lipid production by thraustochytrids using food waste hydrolysate with a higher initial pH (pH7~pH8) adjusted by NaHCO_3_.

## 1. Introduction

Energy consumption and environmental disruption have raised suggestions that biofuel could be an excellent substitute for petrochemical energy due to its clean and sustainable characteristics. Because it contains amounts of organic and inorganic nutrients, food waste has been widely used as feedstock for microbial fermentation in lipid, hydrogen, and methane production [1,2,3,4,5]. In addition, functional chemicals, such as polyunsaturated fatty acids [6], squalene [7] and carotenoids [8], as well as polymer degradable biomaterials such as biobased polyhydroxybutyrate [9], succinic acid [10], plasticizers and surfactants [11], could be synthesized through microbial conversion utilizing food waste. Thus, food waste could be an effective and sustainable substrate for biochemical production, and its bioconversion facilitates environmental protection and alleviates the energy crisis.

Lipid production by oleaginous microorganisms could be exerted utilizing various industrial and agricultural waste products [12,13,14,15,16]. As food waste has increased worldwide, research has increased exponentially, focusing on bioconversion to high value-added substances or biomass energy by microbial fermentation. Bioconversion efficiency for lipids could be influenced by the composition of food waste, lipid-producing characteristics of microorganism strains, environmental factors such as pH, temperature, and cultivation time in the bioprocess [8,17,18,19].

The composition of food waste is complicated and quite different depending on the sources [20,21]. Pretreatment of food waste is a key procedure in the bioconversion process, and can degrade refractory macromolecular nutrients into small absorbable ones, such as glucose and amino acids. Acidic and enzymatic hydrolysis methods are commonly used for pretreatment [15,22,23,24]. Acidic hydrolysis, using sulfuric acid with high temperatures simultaneously, can degrade completely in a short period. Meanwhile, the enzymatic method, known for its advantages of being non-polluting and mild, relies on hydrolysis by various enzymes such as amylase, protease, lipase, and cellulase, and is thus costly.

As the earliest and most studied oleaginous microorganisms, oleaginous microalgae and yeasts from freshwater and marine environments can produce lipids by nature or genetic modification. Furthermore, some protists such as thraustochytrids and labyrinthulids, can synthesize over 20% of lipids, accounting for their own dry weight utilizing an artificial medium or various types of waste, and have exhibited excellent potential for lipid production [7,25,26,27,28]. Among them, the majority of studies focused on the polyunsaturated fatty acid (PUFA) production of thraustochytrids, which can be applied in the food, pharmaceutical, and feed industries. Meanwhile, several concentrated on saturated fatty acids (SFAs) and monounsaturated fatty acids (MUFAs), which can be used for biodiesel production [29,30,31].

Thraustochytrids, a marine heterotrophic oleaginous protist, can utilize sweet sorghum juice [32], corn steep liquor [27], biodiesel-derived crude glycerol [33], aquaculture wastewater [34], cane molasses, and algae-residue [35] for lipid production. Recently, marine protist *Aurantiochytrium* sp. T66 was cultivated on post-consumption food waste hydrolysate, which was then demonstrated to be an excellent low-cost strategy for cultivating thraustochytrids, producing squalene as a byproduct of DHA [7]. In the earliest instances, bread crusts, okara powder, and brewing grain waste had been used as the fermentation substrates for DHA production by thraustochytrids [36]. Moreover, *Schizochytrium mangrovei* utilizing food waste hydrolysate (kitchen waste pretreated by fungus hydrolysis) as substrate exhibited a higher lipid and DHA content (164.9 ± 81.9 mg/g and 40.3 ± 23.5 mg/g) than that in the traditional glucose–yeast extract condition (124.9 ± 43.1 mg/g and 30.2 ± 2.3 mg/g) [21].

The cultivation conditions are important factors affecting the growth and lipid synthesis of thraustochytrids, but there is currently no research on the fermentation of food waste by them from different cultivation conditions (such as carbon, nitrogen, dissolved oxygen, and initial pH). In addition, the pretreatment methods for food waste mainly include physical, chemical, and biological methods, among which the physical method is the most widely used, but it has not been applied to the process of fermenting food waste with thraustochytrids. Therefore, the objective of the present study is to investigate the growth and lipid accumulation capacity of the novel thraustochytrid strain isolated in Northern China under varied carbon, nitrogen, and food waste hydrolysate.

## 2. Materials and Methods

### 2.1. Strain Isolation and Identification

Seawater samples were collected from Western Beach in Qinhuangdao in Hebei Province, Northern China (119.5636° E, 39.9078° N), in July, 2018. The pine pollen method as well as morphological and molecular biology techniques were adopted for thraustochytrid isolation and identification as previously described in Wang et al., 2019 [27]. A thraustochytrid strain HBW10 was obtained and its 18S rRNA sequence was submitted to the National Center for Biotechnology Information (NCBI) database (MZ723876). In addition, an phylogenetic tree was constructed using thraustochytrid sequences blasted in the NCBI (National Center for Biotechnology Information) platform, with *Rhodotorula sinensis* AS2.1389 (KJ708403.1) and *Sporobolomyces phaffii* AS2.2137 (KJ708404.1) as outgroups using Mega 6 software. Furthermore, the thraustochytrid strain HBW10 was deposited in the China General Microbiological Culture Collection Center (CGMCC #3.20948).

### 2.2. Growth Curve and Lipid Production of S. sp. HBW10

A loopful of inoculum of *S*. sp. HBW10 from the solid medium were inoculated into 200 mL seed medium containing 20 g/L glucose, 1.5 g/L peptone, 1 g/L yeast extract, and 33 g/L artificial sea salt, and were cultivated at 28 °C and 150 rpm for 24 h. Then, 5 mL seed cultures were inoculated into 50 mL flasks and cultivated for 7 days in similar conditions to the above. Samples (5 mL) were withdrawn every 2 days from the flasks, and then were centrifuged and washed using sterile distilled water three times. Subsequently, they were stored at −80 °C until further biomass and lipid analysis.

### 2.3. Lipid Production with Varied Carbon and Nitrogen Substrates

The basal medium for both the seed and fermentation cultures was composed of 20 g/L glycerol, 2.5 g/L yeast extract, 0.25 g/L KH_2_PO_4_, and 33 g/L artificial sea salt [27]. Single-factor fermentation experiments were exerted to optimize the carbon and nitrogen sources that could promote the growth and lipid production of *S*. sp. HBW10. The sole carbon, including 20 g/L of glycerol, glucose, xylose, sucrose, and 5 g/L soluble starch, was investigated with no carbon in the medium as control. The sole nitrogen, including 2.5 g/L of yeast extract, peptone, monosodium glutamate (MSG), tryptone, polypeptone, corn steep liquor (CSL), NH_4_Cl and KNO_3_, was studied. Seed inoculum (5 mL) cultivated for 1 day was inoculated into the culture flasks, which were subsequently incubated for 5 days at 28 °C and 150 rpm. Then, samples (5 mL) were withdrawn from the flasks and cryopreserved until further analysis.

### 2.4. Batch Axenic Culture with Food Waste Hydrolysate (FWH)

#### 2.4.1. Preparation of Food Waste Hydrolysate (FWH)

Two types of food waste were collected from two different restaurants in Haigang District, Qinhuangdao City, Hebei Province, China. Type one, mainly containing meat, fat, chicken, and vegetables as well as small amounts of rice and noodles, was collected from a stir-fry restaurant. Type two, mainly containing noodles and rice along with small amounts of vegetables, meat, and fat, was collected from a noodle restaurant. Food waste was carried immediately to the laboratory, and mixed homogeneously before being pretreated with the following procedures. Each 500 g of food waste was immersed in 1000 mL of 3 mol/L (*v*/*v*) sulfuric acid solution at 120 °C for 60 min. The grease was removed after being cooled to room temperature. Then, two types of food waste hydrolysate (FWH1 and FWH2) were obtained by filtrating using a Brinell funnel twice, with a double-layer ordinary filter and a 0.45 μm cellulose acetate filter membrane successively.

#### 2.4.2. Lipid Production with Different Content Levels of FWH

The basal medium for the seed and fermentation culture was composed of 60 g/L glycerol, 15 g/L yeast extract, 0.5 g/L KH_2_PO_4_, and 16 g/L artificial sea salt. A fermentation medium was prepared by adding certain amounts of FWH into the basal medium to reach 50% and 100% content (experimental group), and the basal medium without adding FWH was taken as the control (0% group). Then, the initial pH of the experimental group was adjusted to be equal to that of the control group (pH 6) using NaOH or NaHCO_3_ solution (1 mol/L). The fermentation medium was obtained after autoclaving and cooling to room temperature. Five milliliters of seed culture was inoculated into 250 mL Erlenmeyer flasks containing 50 mL fermentation medium, and was then cultivated for 5 days. For the FWH1 medium, adjusting the initial pH with NaOH, samples were withdrawn every 2 days. Meanwhile, for the FWH1 medium with NaHCO_3_ and the FWH2 medium with NaOH, samples were withdrawn on the fifth day.

#### 2.4.3. Influence of Initial pH on Lipid Production

The initial pH of the culture medium with three content levels of FWH1 (0%, 50% and 100%) was adjusted using 1 mol/L NaOH solution. The initial pH gradients set for the 0% and 50% FWH1 group were pH6.2, pH7, and pH8, and for 100% FWH1 group were pH6.4, pH7, and pH8. After being autoclaved and cooled to room temperature, batch cultures with 50 mL culture medium were incubated for 5 days. Then samples were harvested and prepared for lipid analysis.

For all the experiments above, flask cultures were conducted in a shaker at 28 °C and 150 rpm. Samples withdrawn from the fermentation culture were centrifuged and washed using sterile distilled water three times, and the subsequent biomass samples were stored at −80 °C until further analysis. All the experiments were conducted in triplicate.

### 2.5. Analytical Methods

#### 2.5.1. Biomass Analysis

The biomass samples mentioned above were lyophilized for 48 h and weighted. The biomass was measured in the form of dry cell weight (DCW) by the gravimetric method.

#### 2.5.2. Lipid extraction and analysis

The lipid production was measured by fatty acid methyl esters (FAMEs), which were prepared using the direct transesterification method [37]. The freeze-dried cell pellets were mixed with 2 mL of 4% H_2_SO_4_ in methanol and 100 μL of 1 g/L internal standard (nonadecanoic acid, C19:0) solution. At transesterification reaction was carried out through heating in a water bath at 80 °C for 1 h. One milliliter of water and 1 mL of hexane were added to the mixture, and centrifuged after being vortexed homogeneously. Then the upper hexane layer was filtered through 0.45 μm nylon filters, and the FAMEs in it were analyzed by a gas chromatography Agilent 7890B (Agilent, Santa Clara, CA, USA) equipped with a DB-FastFAME column (60 m × 0.250 mm × 0.25 μm) and a hydrogen flame ionization detector (FID). One μL of the sample was injected in split mode (50:1) with nitrogen as the carrier gas. The temperature of the injector port was set at 250 °C. The oven temperature began at 50 °C and was held for 1 min, followed by programming at 25 °C/min to 175 °C, 80 °C/min to 220 °C and being held for 5 min, then 2 °C/min to 230 °C and being held for 9 min. The FAME peaks were identified by comparing the retention times with those of the 37 FAME standard mixtures (Sigma-Aldrich, Saint Louis, MO, USA). The FAME concentration was quantified by the peak area using an internal standard method.

## 3. Results

### 3.1. Strain Isolation and Phylogenetic Analysis

**Figure 1:**
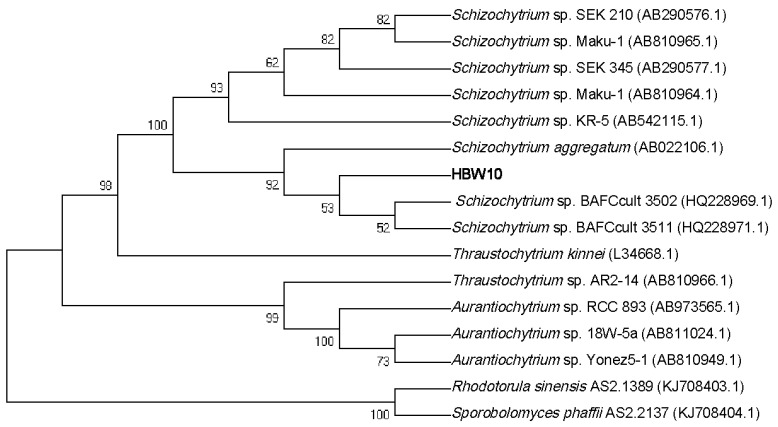
Neighbor-joining tree of HBW10 with *Rhodotorula sinensis* AS2.1389 (KJ708403.1) and *Sporobolomyces phaffii* AS2.2137 (KJ708404.1) as outgroups.

A thraustochytrid strain was isolated from the temperate water column at Western Beach in Bohai Bay, Northern China when a brown tide broke out in July 2019. Microscopic morphological observation was repeated at regular intervals until the pure thraustochytrid strains were obtained. A neighbor-joining phylogenetic tree of 18S rRNA genes was built to classify the evolutionary relationship between the newly isolated thraustochytrid strain and its closest relatives (Figure 1). The phylogenetic tree showed all sequences had a common root and these thraustochytrid sequences were distinguished with two outgroups indicating that they share a common ancestor. In addition, the novel strain HBW10 was grouped into the genus *Schizochytrium*, which was closer to the genus *Thraustochytrium* than *Aurantiochytrium*. Eventually, based on morphological and biological methods, the new strain was identified as *Schizochytrium* sp. HBW10.

### 3.2. Characteristics of S. sp. HBW10 for Biomass and Lipid Accumulation

**Figure 2:**
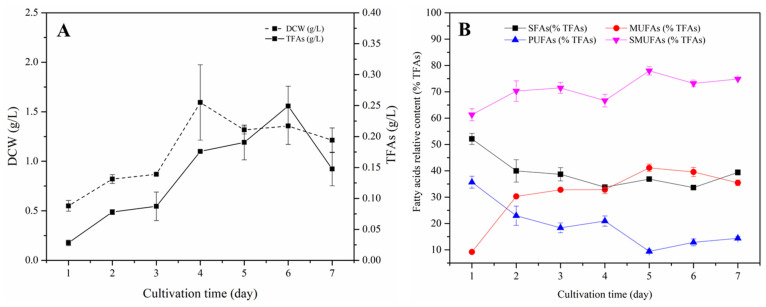
Dry cell weight (DCW), total fatty acid (TFA) production, and fatty acid composition of *S*. sp. HBW10 in the artificial medium at different cultivation stages. (**A**) DCW and TFAs of *S.* sp. HBW10; (**B**) Fatty acid composition of *S*. sp. HBW10.

The biomass and lipid accumulation of *S.* sp. HBW10 were analyzed every two days. The ranges of biomass and total fatty acid (TFA) concentration were 0.55~1.59 g/L and 28.23~249.11 mg/L, and the maximum occurred on the fourth and sixth days, respectively (Figure 2A). In this conventional glucose–peptone medium, the lipids of *S.* sp. HBW10 mainly constituted SFAs and MUFAs, the sum of which accounted for 77.98% of the total fatty acids (TFAs) on the fifth day (Figure 2B), while the PUFAs only made up 9.40% of the TFAs on the fifth day.

### 3.3. Lipid Production of S. sp. HBW10 under Varied Carbon and Nitrogen Substrates

All the carbon and nitrogen sources studied herein promoted the growth and lipid production in *S.* sp. HBW10. The fatty acid composition of *S.* sp. HBW10 in varied carbon and nitrogen sources was mainly SFAs and MUFAs, with a few polyunsaturated fatty acids and C18:2. Furthermore, the fatty acids were predominated by C18:1 and C16:0, followed by C22:6, C18:2, C20:4, C17:1, C18:0, and C17:0, as well as small amounts of C14:0, C16:1, C18:2, C20:0, C20:2, and C20:3.

**Figure 3:**
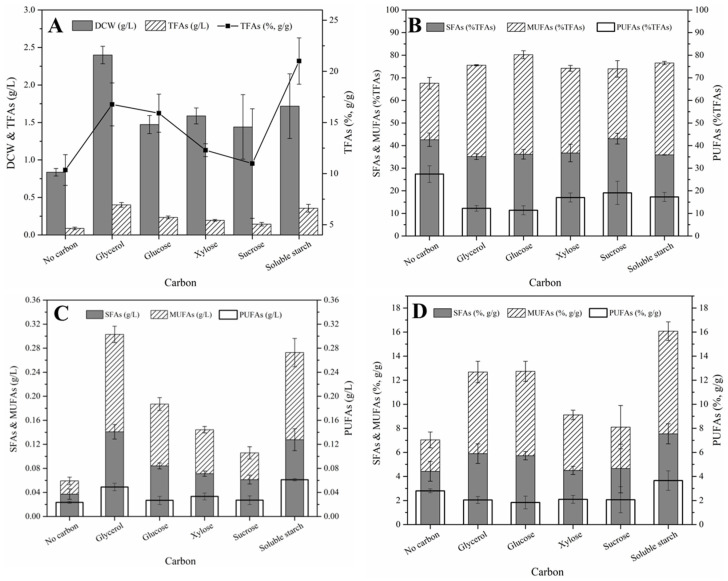
Dry cell weight (DCW), total fatty acid (TFAs) production, and fatty acid composition of *S*. sp. HBW10 with different carbon sources. (**A**) DCW, TFAs concentration and TFAs content; (**B**) The relative content of SFAs, MUFAs and PUFAs in TFAs; (**C**) The concentration of SFAs, MUFAs and PUFAs; (**D**) The content of SFAs, MUFAs and PUFAs.

As for the carbon source, the maximum DCW and TFA concentrations of *S.* sp. HBW10 were observed when glycerol was provided as the sole carbon source, and reached 2.40 g/L and 0.40 g/L separately (Figure 3A), followed by soluble starch, which simultaneously supported fatty acid production with the highest TFAs content (21.01% of the DCW) (Figure 3A). In addition, the concentration and content (%DCW) of SFAs, MUFAs, and PUFAs were generally higher in these two carbon sources among all of those investigated in this study (Figure 3B,C). From this perspective, *S.* sp. HBW10 was speculated to have potential in the treatment of starch-rich waste. In addition, the sum of the relative content of SFAs and MUFAs (SMUFAs) (accounting for the TFAs) was the highest (80.23%) with glucose (Figure 3D), which was considered as an excellent candidate for biodiesel production. Moreover, this was similar to *S.* sp. PKU#Mn4, isolated from mangroves in coastal waters in Shenzhen Province, with higher relative SFA content in glucose [38].

**Figure 4:**
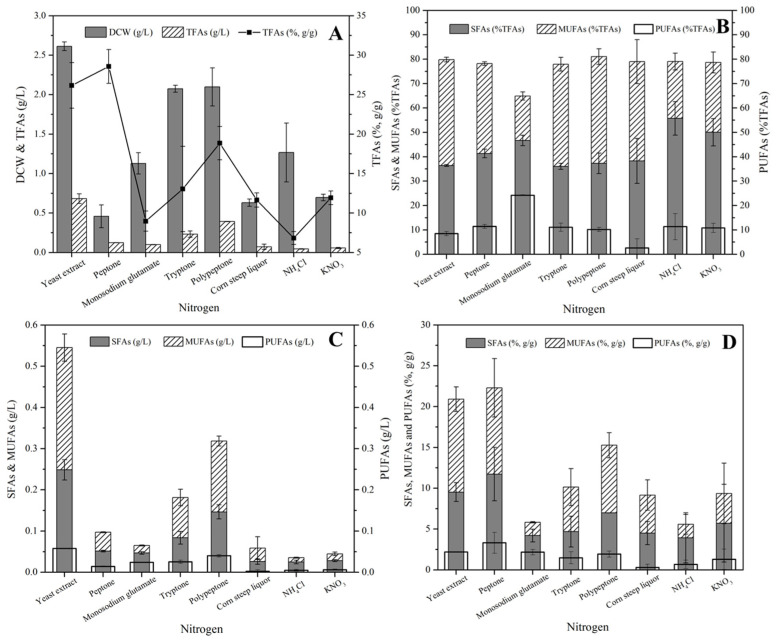
Dry cell weight (DCW), total fatty acid (TFA) production, and fatty acid composition of *S*. sp. HBW10 with different nitrogen sources. (**A**) DCW, TFAs concentration and TFAs content; (**B**) The relative content of SFAs, MUFAs and PUFAs in TFAs; (**C**) The concentration of SFAs, MUFAs and PUFAs; (**D**) The content of SFAs, MUFAs and PUFAs in DCW.

Though both organic and inorganic nitrogen could be utilized by *S.* sp. HBW10, the former generally supported higher fatty acid production (Figure 4A). The maximum DCW and TFA concentrations (2.61 g/L and 0.68 g/L) were obtained with yeast extract as the sole carbon (Figure 4A), accompanied with the highest SFA, MUFA, and PUFA concentrations (0.25 g/L, 0.30 g/L, and 0.06 g/L) (Figure 4B). Meanwhile, the minimum DCW was obtained when grown in peptone, which was contrary to the previous studies that demonstrated peptone to be a better nitrogen for biomass accumulation [32,38,39,40]. However, the highest TFA, SFA, and PUFA content (in the DCW) was observed in peptone (Figure 4A,C). Notably, because of the highest relative content of C20:4 and C22:6, the maximum relative PUFA content (24.19%) was observed in the MSG medium (Figure 4D), while the minimum relative MUFA content was attained in MSG conditions due to the least amount of C18:1 in the total fatty acids, which was twice less than that under other organic nitrogen conditions. Furthermore, compared with organic nitrogen, inorganic nitrogen was more beneficial for SFA conversion in TFAs, because higher relative SFA content was observed in NH_4_Cl and KNO_3_ with the highest C15:0, C17:0, and C18:0 (Figure 4D).

### 3.4. Utilization of FWH as Feedstock for Lipid Production of S. sp. HBW10

Two sources of FWH were added to the fermentation medium at the 0%, 50%, and 100% levels for *S.* sp. HBW10 to investigate its potential for utilizing waste with the initial pH adjusted by NaOH or NaHCO_3_. On the whole, the FWH supplementation into the culture medium had little effect on fatty acid composition, similar to the control group (0% FWH) which mainly included SFAs and MUFAs, as well as a few PUFAs (Table S1), while the only double bond long-chain fatty acid C18:2 varied greatly among different cultivation conditions such as changed sources, food waste content, and carbon dioxide concentration in the experimental group.

Firstly, the TFA concentration and fatty acid composition of *S.* sp. HBW10 grown in FWH1 medium at varied cultivation times were investigated with the initial pH adjusted by NaOH. Except for 100% FWH, the highest lipid concentration occurred on the fifth day, with 0.17 g/L and 0.10 g/L at the 0% and 50% FWH1 levels (Table 1). Similarly, higher relative content levels of SFAs, MUFAs, and PUFAs were also observed on the fifth day. In addition, despite the augment of FWH1 in the fermentation medium suppressing TFA accumulation, a higher TFA concentration was obtained at the 50% FWH1 level with 0.10 g/L on the fifth day (Table 1). Obviously, compared with the control group, the PUFA conversion of the experimental group was greatly inhibited in TFAs in the cultivation period of 5 days.

**Figure 5:**
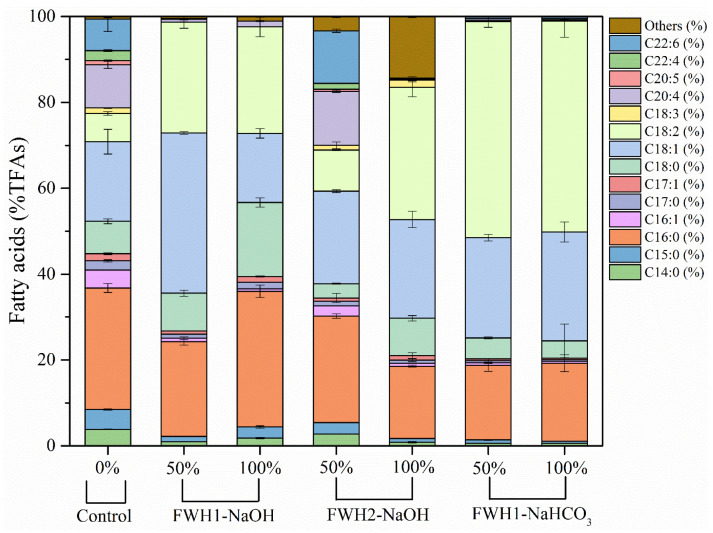
Fatty acid composition of *S*. sp. HBW10 utilizing varied contents of food waste from two different restaurants with initial pH adjusted by NaOH or NaHCO_3_.

Secondly, FWH2 was supplemented to the fermentation medium of *S.* sp. HBW10 to compare the influence of two different sources of food waste on TFA accumulation and fatty acid constitution. Results showed that the TFA concentrations in the FWH2 medium were significantly higher than those in FWH1, and the highest maximum of 0.34 g/L was obtained at the 50% FWH2 level, which was twice and 3.4-fold as much as those at the 0% and 50% FWH1 levels (Table 1). Meanwhile, the relative SMUFA content levels of the former group were lower than those of the latter ones because of the reduction of SFAs (C14:0, C15:0, C16:0, C17:0, and C18:0) and MUFAs (mainly C18:1), separately (Table 2). In addition, the relative PUFA content in TFAs decreased promptly with FWH1 at the 50% level, while *S.* sp. HBW10 accumulated more PUFAs (27.81% of TFAs), especially C22:6 with FWH2 at the same level (Table 1, Figure 5). This indicated that FWH2 would be a better feedstock for PUFA production.

Finally, to explore the effect of CO_2_ pressure on lipid production of *S.* sp. HBW10, NaHCO_3_ was used to adjust the initial pH of the FWH1 medium. The highest TFA concentration of the FWH1-NaHCO_3_ group was observed at the 50% level, and reached 0.20 g/L which was twice as much as that at the 50% level of the FWH1-NaOH group on the fifth day (Table 1). However, compared with the latter one, the relative content levels of SFAs, MUFAs, and SMUFAs of the former group decreased by nearly a third, and that of PUFAs augmented slightly (Table 1), while the most remarkable fatty acid, C18:2, made up half of the TFAs and improved by double in the FWH1-NaOH group (Figure 5). These results indicated an excellent and economical strategy for lipid production industrially using *S.* sp. HBW10 probably stimulated by CO_2_ liberated by NaHCO_3_ degradation in the culture process with the 50% content level of FWH1.

### 3.5. Influence of the Initial pH on the Biomass and Lipid Production of S. sp. HBW10

Flask batch cultivation of *S.* sp. HBW10 was conducted in varied initial pH values of the FWH1 medium to ascertain its effect on the growth and lipid production of *S.* sp. HBW10. As indicated in Table 2, *S.* sp. HBW10 could grow and accumulate fatty acids in both the whole artificial and FWH1 media with varied initial pH values. Except for the 0% group, the biomass and TFA production of *S.* sp. HBW10 enhanced with the increase of the initial pH value, with the maximum at pH8 reaching 4.9- and 1.2-folds (3.80 g/L and 0.12 g/L) compared with that at pH6.2 at the 50% level, and 1.7- and 1.3-folds (2.50 g/L and 0.05 g/L) compared with that at pH6.4 at the 100% level (Table 2). Due to the increase of the initial pH value, the relative content of SFAs largely rose, while that of MUFAs decreased drastically in both the control and experimental groups. However, the relative PUFA content made little change to the whole range of initial pH values. Though these results above exhibited similar variation tendencies both in the control and the experimental groups, the SMUFAs differed. The SMUFA content in TFAs obviously improved in the FWH1 medium and showed little change in the control group with the increase of initial pH value. Generally, it is convenient for the downstream separation of fatty acids in biodiesel production industrially to control higher initial pH values in the fermentation process to stimulate the growth and synthesis of SFAs, simultaneously restraining the synthesis of PUFAs in the FWH medium.

Except for the 50% FWH group, *S.* sp. HBW10 tolerated a large range of initial pH values of the culture medium in the other groups (0% and 100% FWH) (Table 2). The results of the present study indicated that a high initial pH value benefited the biomass and TFA and SFA accumulation of *S.* sp. HBW10 isolated from coastal seawater of Northern China (Table 2). Inversely, *S.* sp. PKU#Mn4, a thraustochytrid strain isolated from mangrove habitats of Southern China, exhibited a declining trend for biomass and TFA and SFA concentration with the initial pH value increasing from 6 to 8 [38]. In addition, the high relative content of SMUFAs in TFAs of *S.* sp. HBW10, reaching up to 97% in the FWH medium, suggested that a high initial pH value of a low-cost FWH culture medium could be a good regulatory strategy for producing SFAs and MUFAs, which can be used as feedstock in biodiesel synthesis.

In summary, an oleaginous thraustochytrid strain was isolated and identified for the first time from Qinhuangdao coastal habitats in Bohai Bay in China. Then the characteristics of lipid production of this thraustochytrid strain were inspected, including the ability to utilize food waste. Firstly, the influence of carbon and nitrogen sources in an artificial medium on lipid production were investigated in order to obtain the optimal condition and reveal its extensive application. Subsequently, food waste from two different restaurants was collected, hydrolyzed, and supplemented into the culture medium to study the effect on TFA production and fatty acid composition. Finally, lipid production under varied initial pH values of a culture medium with different content levels of FWH was investigated. Together, the present study aimed to find a potential strategy to produce lipids using food waste with the newly isolated thraustochytrid strain.

## 4. Discussion

A thraustochytrid strain was isolated from seawater from Western Beach in Qinhuangdao City in Bohai Bay, Northern China when a brown tide broke out. Compared with the isolation process in the same area previously, when no thraustochytrid strains were harvested, it was feasible to acquire it in this study. This might be explained by the phytoplankton bloom which triggered periodic explosive growth of thraustochytrids, a kind of saprophytic protist. Antibiotics were added into the isolation and purification medium for bacteria and fungi elimination as mentioned previously [6]. Notably, more antibacterial drugs were needed for strain isolation of thraustochytrids in temperate habitats than that in tropical waters.

In the present research, *S*. sp. HBW10 could utilize various types of carbon and nitrogen for growth and lipid production, which exhibited excellent potential in the utilization of organic residues and wastes. It is beneficial for the industrial application of *S.* sp. HBW10 to utilize various carbon and nitrogen sources in order to broaden the range of substrate utilization and thus decrease the economic cost. In addition, it exhibited great potential for saturated and monounsaturated fatty acid synthesis, which could be excellent feedstock for biodiesel production. Alternatively, a variety of oleaginous microorganisms, such as microalgae and yeasts, could produce bioenergy, i.e., bio-hydrogen, methyl alcohol, and lipids, cultivated in low-cost and well-sourced substrates, which is beneficial to environment protection and oil production. In addition, our strain *S*. sp. HBW10 was able to produce high-economic-value PUFAs simultaneously, which could possibly be reinforced by adaptive laboratory evolution strategies and genetic engineering for metabolite accumulation [16].

For *S*. sp. HBW10, the relative content of PUFAs in the total fatty acids was obviously less than in the thraustochytrid strains previously isolated from the tropical and sub-tropical areas of China as we reported previously, not to mention the cold habitats [6]. Thraustochytrid strains isolated from a low-temperature environment had been speculated to produce more PUFAs, which had been proved by our previous study [27], as well as those reported elsewhere [41,42,43]. For example, thraustochytrids strains isolated from the cold water of the North Sea region produced more PUFAs reaching up to 47% of TFAs with little effect of the medium type at the screening stage [43]. In addition, compared to thraustochytrid strains isolated from India, Australian thraustochytrids exhibited higher DHA content levels, ranging from 17 to 31% the TFAs, and taking the total omega-3 PUFA content up to 40% of the TFAs [42]. Therefore, further genomic and transcriptome analysis is necessary to reveal the peculiar fatty acid metabolic mechanism of *S.* sp. HBW10 distinguished from other thraustochytrid strains.

It had been demonstrated that lipid production of thraustochytrids could be activated under adverse environments such as nitrogen limitation, low temperature, and oxygen absence, based on which a variety of fermentation strategies had been developed and applied successfully [44,45,46,47]. In the present study, the lipid accumulation of *S.* sp. HBW10 increased on the fifth day due to the decline of dissolved oxygen which was ejected by the constant release of CO_2_ in the fermentation process. In particular, the unsaturated fatty acid C18:2 of *S.* sp. HBW10 reached over 50% of the TFAs in the FWH medium, which provided a possible way to biosynthesize this functional compound for the pharmaceutical industry.

The present study demonstrated that food waste hydrolysate could be utilized by the newly isolated thraustochytrid strain *S*. sp. HBW10 for lipid production. As a whole, *S.* sp. HBW10 has strong adaptability to the nutritional and environmental conditions in the culture medium supplemented with FWH in the present study. Considering the abundant nutrients in food waste including organic and inorganic substances, a further mixed culture of *S.* sp. HBW10 with microalgae in the future seems to be a good strategy to enforce the utilization of food waste, because microalgae prefer inorganic carbon and nitrogen, and utilize CO_2_ for synthesizing organic matter by photosynthesis, while thraustochytrids are inclined to choose organic matters and O_2_ for growth and lipid production. Thus, the effective utilization of food waste and lipid synthesis could be promoted through a synergy effect such as nutrient complementarity, gas exchange (CO_2_ and O_2_), and pH regulation between *S.* sp. HBW10 and microalgae in light conditions.

Lipid production by fermenting food waste by thraustochytrids has important application potential, but many problems and challenges have to be considered in the large-scale application, firstly but not limited to the stable supply of sufficient food waste, which is significant for the stability of lipid production and quality. The pretreatment method of food waste should be environmentally friendly, economical, and convenient. In addition, large-scale production needs to establish a corresponding wastewater and waste residue treatment system to ensure that environmental pollution is controlled. Thus, this process has the advantages of achieving resource recycling, environmental friendliness, and the sustainable production of bioenergy. Simultaneously, it is necessary to consider many technical challenges, economic costs, and public acceptance for lipid production by fermenting food waste.

Considering the high concentration of organic and inorganic matters, food waste hydrolysate has been considered as a reasonable substrate for microorganisms such as algae and yeasts. However, in the present study PUFA always decreased with the increase of FWH content. This could probably be explained by the species-specific responses of microorganisms to these complex molecules from FWH [48]. For example, *Chlorella pyrenoidosa* could not utilize the organic matters from the nutrient sources [12], while the total fatty acid, ARA, and DHA content increased gradually with increasing concentration of FW hydrolysate. To elucidate a specific mechanism, metabolic information from omics techniques is of significance and is still limited currently.

In the present study, we used a newly isolated strain of thraustochytrid to ferment food waste for lipid production. The aim was to provide a cheap and widely available substrate for the fermentation process and demonstrate the feasibility of using food waste for lipid production by thraustochytrids. It would be possible to enhance the economic and environmental value of lipid production through screening for high-yielding thraustochytrid strains, optimization of culture conditions, and pre-treatment methods for food waste, particularly utilizing bioprocessing techniques.

## 5. Conclusions

In conclusion, *S.* sp. HBW10, a marine thraustochytrid strain, was isolated for the first time from seawater at Western Beach in Bohai Bay, Northern China. Process optimization for biomass and lipid production of *S*. sp. HBW10 indicated high tolerance for varied carbon and nitrogen sources. Food waste collected from local restaurants and hydrolyzed by sulfuric acid could be utilized by *S.* sp. HBW10 for lipid production during cultivation periods. The highest TFA concentration was observed in the FWH2 medium with the 50% content level on the fifth day, reaching up to 0.34 g/L. Furthermore, higher initial pH values promoted the growth and SFA bioconversion of *S.* sp. HBW10, achieving nearly 100% of SMUFAs in TFAs in the FWH medium. Moreover, CO_2_ pressure in the culture medium provided by NaHCO_3_ in the fermentation process could be a favorable factor for fatty acid production, especially for C18:2, a functional fatty acid used in the pharmaceutical industry. It is worth noting that the large-scale process of lipid production using food waste still faces some challenges. However, with the advantages of resource recycling, environmental friendliness, and sustainable development, the present study has provided feasible strategies for the economical application of food waste in lipid production by thraustochytrids.

## Figures and Tables

**Table 1:** Fatty acid production of *S.* sp. HBW10 utilizing varied contents of food waste from two different restaurants with initial pH adjusted by NaOH or NaNCO_3_.

Cultivation Time (day)	FWH (%, *v*/*v*)	TFAs (g/L)	SFAs (%TFAs)	MUFAs (%TFAs)	PUFAs (%TFAs)
**Initial pH adjustment of FWH1 medium by NaOH (FWH1-NaOH)**					
1	0	0.11 ± 0.02	57.36 ± 1.39	11.24 ± 1.05	20.01 ± 1.68
1	50	0.06 ± 0.01	45.90 ± 5.69	17.06 ± 3.18	8.32 ± 1.16
1	100	0.04 ± 0.00	55.26 ± 3.83	17.6 ± 1.09	3.43 ± 0.64
3	0	0.16 ± 0.04	47.28 ± 0.08	25.93 ± 0.85	19.61 ± 0.55
3	50	0.06 ± 0.02	43.68 ± 3.32	13.8 ± 0.29	5.20 ± 0.91
3	100	0.05 ± 0.00	45.91 ± 5.85	15.36 ± 5.51	7.65 ± 1.87
5	0	0.17 ± 0.01	46.5 ± 1.39	24.35 ± 3.04	22.02 ± 4.14
5	50	0.10 ± 0.00	34.07 ± 1.56	38.82 ± 0.16	0.85 ± 0.17
5	100	0.04 ± 0.00	54.80 ± 2.73	17.97 ± 1.10	1.30 ± 0.11
**Initial pH adjustment of FWH2 medium by NaOH (FWH2-NaOH)**					
5	0	0.17 ± 0.01	46.50 ± 1.39	24.35 ± 3.04	22.02 ± 4.14
5	50	0.34 ± 0.03	34.61 ± 0.67	24.72 ± 0.92	27.81 ± 0.08
5	100	0.08 ± 0.01	28.06 ± 1.43	24.69 ± 1.28	0.92 ± 0.07
**Initial pH adjustment of FWH1 medium by NaHCO_3_ (FWH1-NaHCO_3_)**					
5	0	0.17 ± 0.01	46.50 ± 1.39	24.35 ± 3.04	22.02 ± 4.14
5	50	0.20 ± 0.02	24.14 ± 2.64	24.38 ± 1.05	0.91 ± 0.26
5	100	0.11 ± 0.01	23.7 ± 1.80	26.15 ± 2.30	0.91 ± 0.25

**Table 2:** Fatty acid composition of *S*. sp. HBW10 utilizing varied contents of FWH1 with different initial pH adjusted by NaOH.

Food Waste Hydrolysate (%, *v*/*v*)	pH	Biomass (g/L)	TFAs (g/L)	SFAs (%TFAs)	MUFAs (%TFAs)	PUFAs (%TFAs)
0	6.2	4.06 ± 0.27	0.17 ± 0.01	46.50 ± 1.39	24.35 ± 3.04	22.02 ± 4.14
0	7	4.07 ± 0.28	0.10 ± 0.01	65.23 ± 5.34	5.30 ± 0.83	29.48 ± 5.46
0	8	4.09 ± 0.09	0.17 ± 0.01	72.07 ± 4.02	4.55 ± 0.16	23.39 ± 3.95
50	6.2	0.77 ± 0.14	0.10 ± 0.02	34.07 ± 0.31	38.82 ± 1.41	0.85 ± 0.02
50	7	3.45 ± 0.0.54	0.11 ± 0.01	88.25 ± 3.14	11.75 ± 3.14	0.00 ± 0.00
50	8	3.80 ± 0.48	0.12 ± 0.01	89.84 ± 3.60	9.31 ± 2.23	0.85 ± 0.00
100	6.4	1.48 ± 0.59	0.04 ± 0.00	54.80 ± 0.40	17.97 ± 0.40	1.30 ± 0.00
100	7	1.92 ± 0.22	0.04 ± 0.00	97.70 ± 2.07	0.00 ± 0.00	2.30 ± 0.07
100	8	2.50 ± 0.18	0.05 ± 0.01	98.61 ± 1.96	0.00 ± 0.00	1.39 ± 0.06

## Data Availability

Data are contained within the article.

## Supplementary Materials

The following supporting information can be downloaded at: https://www.mdpi.com/article/10.3390/microorganisms11112714/s1, Table S1. Fatty acid composition of *S.* sp. HBW10 utilizing varied content of food waste from two different restaurants with initial pH values adjusted by NaOH or NaNCO_3_.
