# Supplementary material for: Lipid Production of Schizochytrium sp. HBW10 Isolated from Coastal Waters of Northern China Cultivated in Food Waste Hydrolysate
Source: Microorganisms. 2023 Nov 6;11(11):2714. doi: 10.3390/microorganisms11112714 (PMC10672807; doi:10.3390/microorganisms11112714)
Supplement: Supplementary file 1 [file microorganisms-11-02714-s001.zip › microorganisms-2682974-supplementary.pdf]

Supplementary material

Lipid Production of *Schizochytrium* sp. HBW10 Isolated from Coastal Waters of Northern China Cultivated in Food Waste Hydrolysate

Xiaofang Li [1], Xinping Yu [1], Qian Liu [1], Yong Zhang [2] and Qiuzhen Wang [1*]

[1] Ocean College, Hebei Agricultural University, Qinhuangdao, Hebei 066000, China

[2] Marine Environment Monitoring Central Station of Qinhuangdao, SOA, Qinhuangdao, Hebei 066002, China

[*] Correspondence: qqzz1990@163.com

Table S1. Fatty acid composition of S. sp. HBW10 utilizing varied content of food waste from two different restaurants with initial pH adjusted by NaOH or $NaNCO_3$.

Fatty acids	0%	FWH1-NaOH		FWH2-NaOH		FWH1-NaHCO3	
		50%	100%	50%	100%	50%	100%
C14:0 (%)	3.83 ± 0.11	0.96 ± 0.04	1.83 ± 0.16	2.78 ± 0.05	0.82 ± 0.17	0.61 ± 0.08	0.56 ± 0.05
C15:0 (%)	4.62 ± 0.14	1.26 ± 0.02	2.56 ± 0.28	2.65 ± 0.06	0.90 ± 0.02	0.77 ± 0.13	0.54 ± 0.05
C16:0 (%)	28.32 ± 1.04	22.07 ± 0.85	31.65 ± 1.43	24.80 ± 0.51	16.84 ± 0.22	17.41 ± 1.45	18.17 ± 1.95
C16:1 (%)	4.17 ± 0.04	0.77 ± 0.31	0.59 ± 0.06	2.38 ± 0.03	0.65 ± 0.02	0.56 ± 0.20	0.47 ± 0.03
C17:0 (%)	2.18 ± 0.26	0.94 ± 0.02	1.53 ± 0.05	1.06 ± 0.02	0.79 ± 0.39	0.44 ± 0.06	0.37 ± 0.06
C17:1 (%)	1.66 ± 0.21	0.73 ± 0.02	1.32 ± 0.13	0.78 ± 1.09	0.99 ± 0.69	0.45 ± 0.06	0.28 ± 0.02
C18:0 (%)	7.54 ± 0.57	8.83 ± 0.73	17.23 ± 1.06	3.33 ± 0.11	8.72 ± 0.64	4.89 ± 4.24	4.05 ± 3.91
C18:1 (%)	18.53 ± 2.87	37.32 ± 0.31	16.06 ± 1.09	21.56 ± 0.31	23.04 ± 1.94	23.38 ± 0.80	25.40 ± 2.35
C18:2 (%)	6.56 ± 0.44	25.83 ± 1.44	24.86 ± 2.34	9.56 ± 0.16	30.73 ± 2.20	50.31 ± 1.35	49.11 ± 3.82
C18:3 (%)	1.30 ± 0.10	0.00 ± 0.00	0.00 ± 0.00	1.13 ± 0.76	1.69 ± 0.39	0.23 ± 0.01	0.19 ± 0.21
C20:4 (%)	10.05 ± 0.83	0.63 ± 0.55	1.30 ± 0.11	12.55 ± 0.25	0.22 ± 0.31	0.42 ± 0.06	0.31 ± 0.03
C20:5 (%)	0.97 ± 0.13	0.00 ± 0.00	0.00 ± 0.00	0.50 ± 0.01	0.00 ± 0.00	0.00 ± 0.00	0.00 ± 0.00
C22:4 (%)	2.31 ± 0.20	0.00 ± 0.00	0.00 ± 0.00	1.35 ± 0.06	0.00 ± 0.00	0.00 ± 0.00	0.00 ± 0.00
C22:6 (%)	7.39 ± 2.90	0.22 ± 0.38	0.00 ± 0.00	12.27 ± 0.40	0.24 ± 0.34	0.40 ± 0.01	0.41 ± 0.02
Others (%)	0.57 ± 0.26	0.44 ± 0.10	1.07 ± 0.13	3.30 ± 0.30	14.37 ± 0.31	0.27 ± 0.06	0.13 ± 0.04
